# Combined Intravitreal Dexamethasone Implant And Micropulse Yellow Laser For Treatment Of Anti-VEGF Resistant Diabetic Macular Edema

**DOI:** 10.2174/1874364101711010164

**Published:** 2017-07-21

**Authors:** Ahmed Hosni Abd Elhamid

**Affiliations:** Ophthalmology Department, Ain Shams University Hospital, Cairo, Egypt

**Keywords:** Diabetic macular edema, Ozurdex Intravitreal dexamethasone implant, Subthreshold micropulse laser, Central macular thickness, Best corrected visual acuity, Anti-VEGF

## Abstract

**Purpose:**

To report the efficacy and safety of combined intravitreal dexamethasone implant and micropulse laser for anti-VEGF resistant diabetic macular edema.

**Patients and Methods:**

Prospective, non-controlled study that was conducted for twenty eyes with center-involved diabetic macular edema not responding to anti-VEGF therapy. Ozurdex intravitreal implant was injected to all eyes with subsequent micropulse yellow laser one month after the injection. All eyes were followed up after one, three, four, six, nine and twelve months. The primary outcome measure is the change in best corrected visual acuity (BCVA) after one year and secondary outcome measures are central macular thickness (CMT) change and safety of both dexamethasone implant and micropulse laser. Reinjection was done for those eyes with recurrent edema.

**Results:**

The mean age was 58.8 ±7.94 years. The mean BCVA was 0.6± 0.14, 0.57 ±0.12, 0.51±0.15, 0.59±0.12, 0.6± 0.12 and 0.59±0.14 after one, three, four, six, nine and twelve months in comparison to 0.45± 0.14 as initial BCVA [SS,P<0.05]. The CMT was 302.5±30.01, 330.6±20.24, 357.6±32.15, 285.4±19.95, 292.9±25.07 and 285.2±14.99 after one ,three, four ,six , nine and twelve months µm in comparison to initial CMT of 420.7 ±38.74µm [HS, P<0.01]. Cataract occurred in 6 eyes from 14 phakic eyes (42.8%). Transient ocular hypertension occurred in 6 eyes (30%). Reinjection was done for eight eyes (40%).

**Conclusion:**

Intravitreal dexamethasone implant and micropulse laser are both effective and safe treatment options for anti-VEGF resistant diabetic macular edema.

## INTRODUCTION

Diabetic retinopathy is a well-known cause of vision loss, diabetic macular edema (DME) is the commonest cause of vision loss in diabetic retinopathy [1]. The pathogenesis of DME is multifactorial; inflammation represents one of the leading arms in disease development and progression. It has been observed that many inflammatory mediators are released from hypoxic retina, such as cytokines including interleukin (IL)-6, IL-8, prostaglandins in addition to vascular endothelial growth factor (VEGF), all these factors can cause loss of both pericytes and endothelial cells resulting in leakage [2].

Many options are available for treatment of DME like focal/grid laser photocoagulation and pharmacologic therapy including intravitreal injection of various anti-vascular endothelial growth factor (VEGF) agents and corticosteroids [3].

Intravitreal injection of corticosteroids can reduce macular edema by stabilization of capillary walls and also by preventing release of leucocytes, VEGF, prostaglandins and other pro inflammatory cytokines [4].

Dexamethasone is one of the potent anti-inflammatory steroids. It is about six times more potent than intravitreal triamcinolone acetonide. Single intravitreal injection of triamcinolone either 4 or 1.2 mg does not provide constant level in the vitreous cavity and also carries the risk of high incidence of glaucoma and cataract. Long acting intravitreal dexamethasone implant (IDI) 0.7 mg (Ozurdex; Allergan, Irvine, CA, USA) is a sustained release biodegradable implant of poly lactic-*co*-glycolic acid (PLGA) which is approved for use in DME and retinal vein occlusion [5, 6]. The Ozurdex implant is a rod in shape, measured 0.46 mm in diameter, 6 mm in length and is injected through 22 G applicator. The implant lasts for up to 180 days inside the vitreous cavity after injection with slow progressive biodegradation that allows the presence of constant amount of the drug in the vitreous cavity for about four months without having the effect of large drug dose immediately after injection. In a large randomized clinical trial in patients with DME, *Boyer et al.* found that the IDI 0.7 mg met the primary efficacy endpoint for improvement in BCVA at three years with an acceptable safety profile [7]. Some small retrospective reports have also noted success of IDI for DME and recalcitrant macular edema of other causes [8, 9].

The Early Treatment Diabetic Retinopathy Study (ETDRS) reported favorable results of laser photocoagulation for treatment of diabetic macular edema with 50% reduction in visual loss after three years of follow up [10]. Despite the satisfactory results achieved, scars induced by conventional laser may enlarge with subsequent significant scotomas and visual field defects [11].

It was observed that the optimum therapeutic effect produced by laser to treat DME is achieved by still-viable and activated RPE cells by heat stimulation at sub-thermal elevations not by RPE killed cells through thermal heat produced by conventional laser treatment. Advances in laser technology have led to the development of selective photocoagulation for the RPE via the sub threshold micropulse laser method. This is designed to target and activate the RPE, while having a minimal effect on the sensory retina and choroid. The idea of the micropulse technology is to divide the pulse envelope into100 micropulses, each micropulse has on and off time, the micropulse on time will be specified according to the used duty cycle (5-15%). This will produce an invisible reaction which is detectable only with microscopy and histology and will be sufficient to produce an RPE-confined photothermal effect with sparing of the neurosensory retina. The solid state 577-nm yellow laser light is mainly absorbed by oxyhemoglobin and melanin with negligible xanthophyll absorption and low intraocular light scattering and pain. It is theoretically suited to the micro pulse technique aimed at RPE cells with minimal effect on the sensory retina and aids in treatment very close to the fovea. It was first described in *1997 by Friberg and Karatza* [12] as laser therapy for DME. Many studies showed improvement of BCVA and reduction of CMT after micropulse laser treatment for DME [13-16].

The purpose of this study is to report the efficacy and safety of combined intravitreal dexamethasone implant and micropulse laser for anti-VEGF resistant diabetic macular edema.

## PATIENTS AND METHODS

Prospective, non-comparative study that was conducted for 20 patients. Inclusion criteria were age above 18, any sex, center- involved diffuse DME, BCVA ranging from 0.8 to 0.1. All the eyes had persistent DME after at least three consecutive injections of anti-VEGF agents with CMT ≥ 300 um on initial OCT examination. Last intravitreal injection was done at least three months before Ozurdex IDI injection.

Exclusion criteria included the presence of macular scar, foveal hard exudates, epiretinal membrane (ERM), proliferative diabetic retinopathy (PDR), vitrectomized eyes, disrupted IS/OS junction, glaucoma or ocular hypertension. Patients with glycosylated hemoglobin (HbA1c)> 10 are also excluded from the study.

All patients had underwent complete ophthalmic examination starting from BCVA assessment using Snellen chart, slit lamp examination, IOP measurement by GOLDMANN applanation tonometry, indirect ophthalmoscopy and bio microscopy. OCT (TOPCON Corporation, Tokyo, Japan), 3D macula scan was done within one week from Ozurdex IDI.

The study was done under the declaration of tenets of Helsinki. Thorough explanation of the disease with its pathogenesis together with the advantages and disadvantages of IDI in comparison to switching to other anti-VEGF agent was done. Informed consent was signed by all patients.

The intravitreal injection technique of IDI was as follows: Sterilization and drabbing, irrigation of the conjunctival sac with diluted betadine solution 5% followed by injection of the implant 4mm from the limbus for phakic eyes and 3 mm for pseudophakic eyes in the inferotemporal quadrant through the provided 22 gauge injector. Topical antibiotic drops are given to all eyes 4times /day for 10 days.

All eyes were examined after one day, one week, two weeks, one month and every month for one year after injection with special attention to the BCVA, IOP, lens status and change in macular edema, OCT was done at one, three, four, six, nine and twelve months after injection.

Micropulse yellow IQ 577nm laser (Iridex Corporation, Mountain View, CA, USA) was done one month after IDI injection. The Area Centralis lens (laser spot size magnification 0.94) was used for all eyes with power of 400 mw, 200μm spot size and the pulse envelope duration of 200 ms. 5% duty cycle was chosen after activation of micropulse mode. The whole area of edematous macula was treated with variable number of confluent zero spacing shots applied through 7x7 grids. Care was taken to begin treatment outside the foveal avascular zone and to treat the fovea if there is no visible reaction. If there was any visible reaction treatment was stopped and the power was decreased till no visible reaction.

The primary outcome measure was not is BCVA change after one year. Secondary outcome measures were change in CMT after one year and safety of both IDI and micropulse laser.

Reinjection is done after four months from the first injection if there is persistent edema as evidenced by CMT above 300 um or recurrence of edema (increase of 50 um in CMT in comparison to the previous measurement). The reinjection is also followed one month later by another session of yellow micropulse laser photocoagulation. Safety was assessed by monitoring changes in IOP and development or progression of cataract. IOP elevations of ≥5 mmHg from baseline measurement were considered to be steroid induced ocular hypertension.

The number of intravitreal injections and the average period between injections were recorded during the follow-up visits. Phacoemulsification and IOL were done for those patients who developed significant cataract during the study period.

### Statistical Analysis

The raw data were entered into Excel spreadsheets. The Statistical Package for the Social Sciences, version 21 (IBM Corporation, Armonk, NY, USA) was used for analysis. Patient characteristics are expressed as mean ± standard deviation. Paired Student’s *t*-test is used to analyze the BCVA and CMT (Table **1**).

## RESULTS

### BCVA Changes

**Chart. (1):**
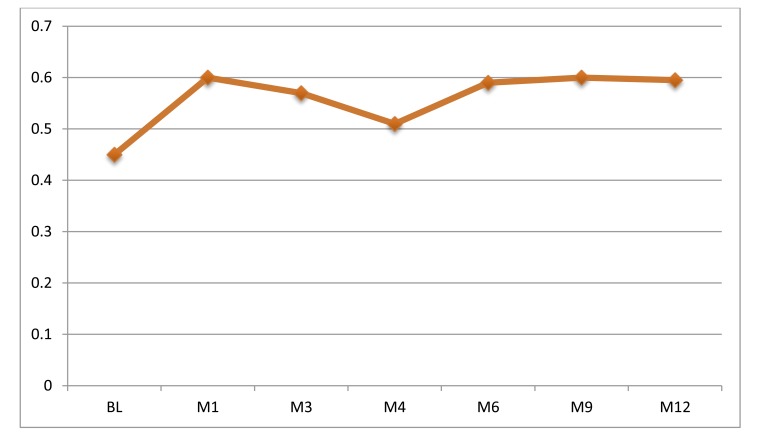
Mean BCVA changes along the study follow up periods, BL = baseline, M = month.

The mean BCVA was 0.6± 0.14, 0.57 ±0.12, 0.51±0.15, 0.59±0.12, 0.6± 0.12 and 0.59± 0.14 after one, three, four,six, nine and twelve months in comparison to 0.45± 0.14 as initial BCVA [SS,P < 0.05] **Chart (1)**.

The final BCVA was improved in 15 eyes (75%), stable in five eyes (25%). 13 eyes (65%) had gained more than two lines improvement in BCVA.

### CMT Changes

**Chart. (2):**
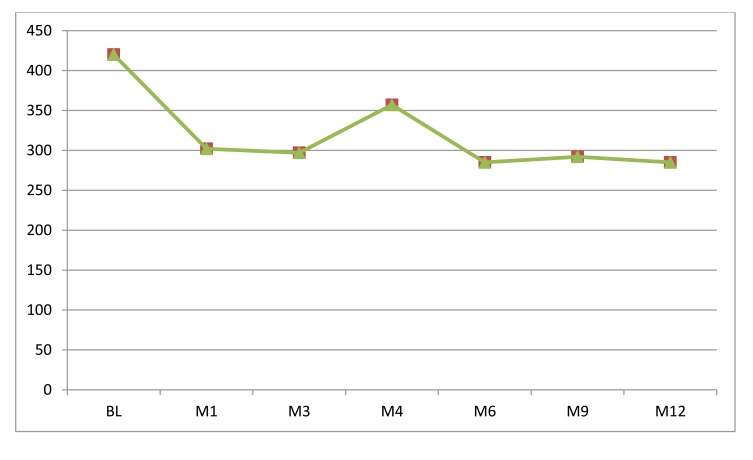
Mean CMT changes along the study follow up periods. BL=baseline, M=month.

As shown in **Chart (2)**, the mean CMT was 302.5±30.01, 330.6±20.24, 357.6±32.15, 285.4±19.95, 292.9±25.07 and 285.2±14.99 after one ,three, four ,six , nine and twelve months µm in comparison to initial CMT of 420.7 ±38.74µm [HS, P<0.01]. The mean final CMT has been decreased by 32.3% in comparison to the initial CMT.

### Complications

Ten eyes (50%) had subconjunctival hemorrhage related to injection site. Cataract occurred in 6 eyes from 14 phakic eyes (42.8%). Two eyes (10%) had mild vitreous hemorrhage that was cleared spontaneously. Intraocular inflammation, intraocular bleeding, hypotony from wound leakage and retinal detachment did not occur in any eyes.

Ocular hypertension occurred in 4 eyes (20%) after one to two months from injection. The range of IOP was from 25-35 mmhg. Topical antiglaucoma treatment was effective in managing all eyes for two months after injection, No eyes needed glaucoma surgery. **Table (2)** shows different values of mean IOP along the follow up visits.

Eight eyes (40%) needed second IDI injection; two eyes (10%) needed third injection. Each injection is followed by one session micropulse laser photocoagulation. The mean number of injections during one year is 1.5 and the mean interval time for the second injection is 17 weeks. The mean time for the third injection is 37 weeks. The average number of laser spots was 431± 87.

## DISCUSSION

DME is the major cause of gradual vision loss in diabetic retinopathy patients. Many therapeutic options are available now-a-days for treatment of DME, among the most important options is intravitreal anti-VEGF therapy. Some eyes are poor responders for anti-VEGF therapy. Recent post hook analysis for patients enrolled in protocol I reported that patients who responded poorly after three consecutive ranibezumab injections are less likely to respond well after one and three years (the mean gain was only 3.00 lines after three years of monthly injections for those who gained <5 letters after three initial consecutive injections and only 28% of them responded well *i.e.* >10 letters after three years of monthly injection) [17], so switching to intravitreal steroid implant could be advisable after the initial poor response to anti VEGF to decrease the burden of repeated injections without expected considerable response.

Dexamethasone is a potent steroid but it has short half-life inside the vitreous cavity (5.5 hours) so it is used infrequently. To extend the duration of activity of dexamethasone, an intravitreal sustained release form was developed which is Ozurdex IDI that was approved as biodegradable implant for treatment of many retinal vascular disorders. The dose of IDI is 0.7 mg within NOVADUR solid polymer drug delivery system (Styrolution; Aurora, Illinois USA). This system is gradually and slowly biodegradable into lactic and glycolic acids which could be eliminated by the eye as carbon dioxide and water [6].

It is noted from this study that there is improvement in final BCVA in 15 eyes (75%) after 12 month follow up period. 13 eyes (65%) had gained more than two lines. These results are comparable to *Boyer et al.* (BCVA was improved in 65%) [7]. *Callanan et al.* [18] showed that 27.8% of the eyes gained more than 10 letters of BCVA (ETDRS chart) after combined 0.7 mg IDI with laser treatment. *Zhioua et al.* [19] reported in his study significant improvement in BCVA and CMT after IDI in ranibizumab resistant DME patients.

The study showed that both efficacy parameters improved from the first month following injection which is related mainly to the therapeutic effect of the drug in the vitreous (by that time the laser effect could not be established). Recurrence of DME occurred in 8 eyes (40%) as evidenced by increase in CMT, the recurrence occurred four months after injection which could be attributed to decrease in vitreous concentration of the drug at that time below the therapeutic effect. *Totan et al.* [20] showed that ozurdex IDI resulted in improvement of both BCVA and CMT of DME patients who were resistant to bevacizumab injection, the improvement was evident only for three months with recurrence from three to six months. The rate of recurrence in this study is considered relatively low (second injection was given for 40%) in comparison to other studies as *Totan et al.* [20] (83%), *Escobar et al.* [21](69.4%), the explanation for that may be attributed to the synergetic effect of micropulse laser with Ozurdex implant, however to prove this synergetic effect there should be another study that includes comparative group in which IDI was only used without micropulse laser.

Cataract is the most common complication that occurred in this study [6 out of 14 phakic eyes (42.8%)]; uneventful phacoemulsification was done for all eyes that had significant cataract before the time for next vision assessment so the measurement of BCVA is not affected. The rate of cataract formation is less than intravitreal fluocinolone in FAME study [22] which was 87.2%, 80% in high and low dose, respectively. Ocular hypertension occurred in 20% of eyes and none of the study eyes required glaucoma surgery which is comparable to *Matoniti et al.* [23] (13.2%) and *Kidde et al.* [24] (15.1%). The incidence of steroid induced ocular hypertension is less than other intraocular steroids; *Elaraoud et al.* showed that 4.8% of eyes treated with low dose intravitreal fluocinolone implant and 8.1% of those treated with high dose implant needed incisional glaucoma surgery. The explanation of fewer incidences of steroid induced glaucoma and cataract with dexamethasone in contrast to triamcinolone and fluocinolone is that dexamethasone is less lipophilic and does not accumulate in trabecular meshwork and lens fibers.

Anti-VEGF medications are considered to be the first-line therapy for DME. However, in partial responders to anti-VEGF treatment or in pseudophakic patients, steroid implants could be a useful option. Both drugs are working well with DME but with fewer injections regarding IDI as evidenced by BEVORDEX study [25] which is a randomized phase ɪɪ trial that compared intravitreal bevacizumab (1.25mg) with IDI (0.7 mg) and found no statistically significant difference in the number of eyes that gained ten or more lines of vision (40% in bevacizumab group versus 41% in IDI). CMT reduction was statistically more in IDI group than bevacizumab group. The average number of injections was more in bevacizumab group (8.6 over 1 year in contrast to 2.7 for IDI). The incidence of cataract was definitely more in the IDI group. Another study done by *Maturi et al* [26]. to evaluate the effect of adding Ozurdex implant to bevacizumab in comparison to bevacizumab alone showed that there was no significant difference in the BCVA but there is significant reduction in CMT in combination group with fewer injections of bevacizumab in combination group than the bevacizumab alone group.

In fact, DME is multifactorial disorder. One important arm in the pathogenesis of DME is the inflammatory component, Dexamethasone is strong anti-inflammatory and antiedema drug that is well-known to down regulate not only VEGF but also other inflammatory cytokines which may explain the proper response to IDI in cases of resistance to anti-VEGF agents, besides that it reduces leukocytosis and decreases vascular leakage.

Micropulse is a laser modality that divides a continuous stream of laser into a number of short bursts separated by pauses (off time). According to the selected duty cycle, the laser stays on only 5%–15% of the time, thus generating less heat with subsequent less damage to the retina than continuous-wave photocoagulation. Subthreshold micropulse laser emission without a visible burn endpoint appears to reduce the risk of structural and functional retinal laser damage. Micropulse laser causes stimulation of a biological response that restores the proper pump function of RPE cells, resulting in enhanced and rapid absorption of subretinal and intraretinal edema fluid. The 577 nm yellow laser is ideal for diseases in which the primary pathology is in the RPE. It is highly selective for the RPE cells and, on the other hand, it is poorly absorbed by the foveal xanthophyll pigments, hence the effects are localized to the RPE and protecting the fovea.

Previous studies have reported the excellent effect of micropulse diode laser treatment on DME in terms of improved BCVA and decreased thickness on OCT [13, 27]. Pei-Pei et al.[28] compared subthreshold with threshold laser grid treatment for patients with DME using a green 532-nm PASCAL system. They found that the mean BCVA and the CMT improved in both the subthreshold group and the threshold group and there was no statistically significant difference between the two groups. *kwon et al* [14] reported improvement of BCVA from 0.5 to 0.4 (log MAR) after micropulse yellow 577nm laser treatment for DME, *Mahmoud [15] and Latlaska *et al*. [16]* also showed improvement in BCVA and reduction in CMT for DME patients treated with micropulse yellow laser. It is clear that micropulse treatment is suitable for mild cases of DME with CMT from 300-350 um which is ideal after initial improvement with either anti-VEGF or IDI.

The limitations of this study are the small number of the eyes included in the study and the lack of comparative group for which IDI was used without micropulse laser treatment to prove the additive effect of micropulse treatment to IDI.

In conclusion, IDI with yellow laser micropulse treatment is effective in treating DME as evidenced by improvement in BCVA in 75% of the eyes as well as significant reduction in CMT. Cataract is the most common adverse effect of IDI which can be managed uneventfully by phacoemulsification with regain of vision. Combined IDI and micropulse laser could be a suitable treatment option for non VEGF mediated DME or even to decrease the burden of anti-VEGF injection.

## Figures and Tables

**Fig. (1):**
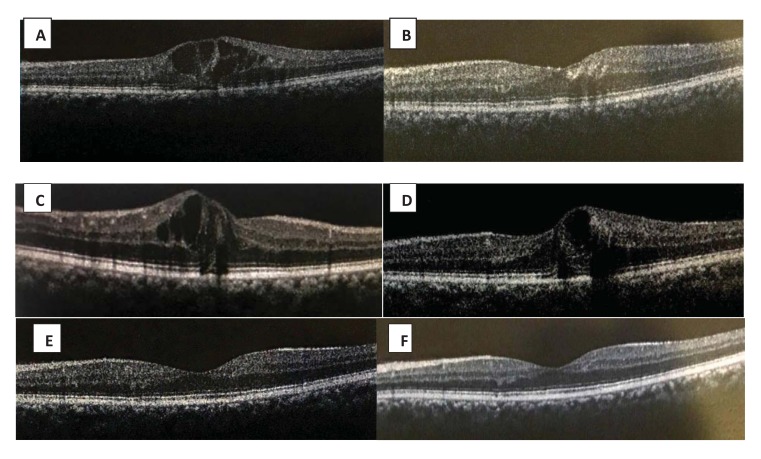
(A) Initial OCT of one patient showing DME, (B) Shows improvement of CMT one month after IDI with micro pulse laser, (C) Recurrent DME after three months, (D) Persistence of DME after four months, (E) OCT done after 6 months with improvement of CMT after second IDI and laser, (F) Final OCT after 12 months with complete resolution of DME.

**Table:** Table **1**. shows the initial characteristics of the patients.

***Initial characteristics***	
***Mean Age***	58.8 ±7.94 years.
***Sex***	Ten were males and ten were females.
***Mean Initial BCVA***	0.45 ±0.14
***Mean Initial CMT***	420.7 ± 38.74um
***Lens status***	14 were Phakic and six were Pseudophakic
***Mean Initial IOP***	17.6 mmhg .
***Mean Duration of DM***	14.4 years.
***Previous treatment***	Laser None Anti VEGF 20(100%) Intravitreal steroids None
***Severity of NPDR***	Mild NPDR 14eyes Moderate NPDR 6 eyes

**Table 2:** Mean IOP values not changes along follow up periods. BL = baseline, M= month, all IOP values are in mmhg.

	BL	M1	M3	M4	M6	M9	M12
**Mean IOP**	17.6	19.5	16.7	16.8	19.4	15.4	17.2
